# Enhancing Communication Among Patients with Cancer, Caregivers, and Extended Family: Development of a Communication Module

**DOI:** 10.3390/ijerph22040541

**Published:** 2025-04-01

**Authors:** Stephanie D. Torres-Marrero, Carled Argüelles-Berrios, Ninoshka Rivera-Torres, Lianel Rosario-Ramos, Alondra De Lahongrais-Lamboy, Normarie Torres-Blasco

**Affiliations:** 1 School of Behavioral and Brain Sciences, Ponce Health Sciences University, Ponce 00716, Puerto Rico; carguelles23@stu.psm.edu (C.A.-B.); nirivera23@stu.psm.edu (N.R.-T.); lrosario21@stu.psm.edu (L.R.-R.); 2 Ponce Research Institute, Ponce 00716, Puerto Rico; adelahongrais@psm.edu; 3 Liberal Arts Department, Ana G. Méndez University, Ponce Campus, Ponce 00716, Puerto Rico

**Keywords:** communication, cancer, patients, caregivers, extended family

## Abstract

(1) Background: Coping with a cancer diagnosis can be a challenging process, in which patients and caregivers often require family support. For an adequate support network, there must be clear communication among patients, caregivers, and family members. However, the literature has not adequately discussed communication interventions with extended family members. Our research team identified the need to incorporate communication strategies among patients, caregivers, and extended family. For this reason, we aimed to develop a communication module for patients with cancer, caregivers, and extended family members. (2) Methods: We conducted two steps to address the study’s objective: (a) a narrative literature review to identify communication strategies or interventions and (b) a data triangulation with the narrative literature review findings, the primary study findings, and the cultural expert expertise. (3) Results: The module content included learning what to say and improving general communication. Within the content were communication strategies such as prompt lists, priority lists, methods of telling others, delegating, seeking support, and others. (4) Conclusions: Enhancing the communication among patients with cancer, caregivers, and extended family promotes adequate family support. Nevertheless, future studies should explore the acceptability and implementation strategies of protocols focused on communication.

## 1. Introduction

Chronic diseases represent a significant and growing public health challenge worldwide, impacting millions of individuals. These conditions, characterized by a gradual progression, prolonged duration, lifestyle changes, and ongoing medical attention, encompass a wide range of ailments, such as diabetes, cardiovascular diseases, chronic respiratory diseases, and cancer [1]. Effectively managing chronic diseases necessitates a multifaceted approach involving continuous medical care, lifestyle modifications, and psychosocial support to enhance patients’ quality of life [2]. The pervasive nature of chronic illnesses not only exacts a heavy physical toll on patients but also profoundly affects their psychological and emotional well-being [3]. Moreover, the burden extends to familial and social dimensions, necessitating cohesive support structures and robust communication networks to mitigate the adverse effects and facilitate optimal management. An example of one of the most prevalent diseases globally that illustrates the multifaceted burden of chronic illness is cancer [4].

Coping with a cancer diagnosis can strain interpersonal relationships, particularly among family members [5]. Family relationships often deteriorate due to the impact that cancer has on patients’ lives [6]. As part of the cancer experience, patients tend to isolate themselves, keep their emotions or thoughts private, and may not share the cancer journey with others [5,7]. In addition to emotional and psychological factors, cultural values play a significant role in how patients manage family dynamics during their illness. An example of this cultural value is familism, a cultural value prominent in Hispanic and Latino cultures, which places the family as the central unit of emotional and social life [8,9]. In the cancer context, familism may influence how patients with cancer communicate their illness to their families [10]. Familism often leads patients to feel a greater sense of responsibility to share their condition with family members, not only as a personal need but also as part of a cultural expectation to maintain a close family unit and mutual support during times of crisis [11]. While this cultural context fosters open communication with family, it can also pressure patients to share more than they may feel comfortable with. Thus, while familism can enhance family support throughout the cancer journey, it also recognizes that communication and seeking family support is a patient’s personal decision.

Despite the challenges of coping with a cancer diagnosis, the evidence suggests that having social network support, specifically from family members, plays an essential role in managing the illness. For many Hispanic and Latino patients, family members are not only a source of emotional support (such as being right there for patients and understanding their decisions and wishes) but also active participants in patients’ medical decision-making [9,12,13]. The role of family members is significant in Hispanic and Latino communities, where studies have highlighted the need to include family members as an immediate network of support during the cancer journey [14]. This support can come from both immediate family (parents, sons, or daughters) and extended family members. The extended family refers to family members, such as grandparents, cousins, aunts, and uncles [15]. Also, despite not being blood relatives, friends can be considered family members due to their close relationship with patients.

Effective communication between patients with cancer and family caregivers is vital to managing family tension and coping mechanisms [16]. Although the patient may benefit from assertive communication with extended family members during the cancer journey, cancer care settings and psychosocial services do not adequately address this aspect of communication. There is often a need for information about the cancer diagnosis among family members due to the lack of communication between patients and their families [6]. Additionally, most communication interventions focus on the caregiver as part of the family or on immediate family members, leaving a gap in interventions that promote communication between patients with cancer, caregivers, and extended family members. Despite the limited literature, our research team found that patients, caregivers, community leaders, and healthcare providers recognize the need to incorporate communication strategies among extended family members [17].

Effective communication among patients with cancer, caregivers, and the extended family is crucial for managing the illness, highlighting the need for interventions that incorporate communication strategies to strengthen family social networks. This study aimed to develop a communication module that enhances communication between patients, caregivers, and extended family members. To address the study objective, we first conducted a narrative literature review to identify communication strategies. Additionally, we conducted a data triangulation with the primary study’s findings, the narrative literature review findings, and the cultural expert’s expertise.

## 2. Materials and Methods

We conducted two steps to develop the communication module: (a) a narrative literature review and (b) a data triangulation. First, we performed a narrative literature review to qualitatively review communication strategies and interventions that may help patients and caregivers communicate effectively with extended families. A narrative literature review allowed us to examine various articles and sources in this area, despite limited literature on communication involving extended family members. Second, we conducted a data triangulation to integrate the primary study findings with the narrative review findings based on the expertise of a cultural expert. The ethical approval for this study corresponds to the primary study (IRB #2206107691A002). Specifically, the narrative and triangulation did not involve direct interaction with participants or handling primary study data. We relied exclusively on the published findings from the primary study and reviewed literature.

### 2.1. Narrative Literature Review

#### 2.1.1. Eligibility Criteria

The eligibility criteria included articles that focused on assertive communication strategies or training between patients and extended family members and articles that mentioned family communication as a component of promoting well-being while coping with cancer. Eligible publications included studies, meta-analyses, systematic or narrative reviews, and websites of official health and cancer support organizations in English or Spanish.

#### 2.1.2. Exclusion Criteria

The exclusion criteria encompassed articles that (1) did not include extended family members beyond the primary caregiver or (2) included family members without clearly specifying whether they were acting as caregivers, other relatives, or both. In addition, articles published before 2010 were excluded, while no date restrictions were applied to online sources.

#### 2.1.3. Information Sources

To identify the articles that met our criteria, we used the following databases: PubMed, EBSCOhost, Scielo, Redalyc, and Google Scholar. We used these databases due to their credibility and accessibility to our research members. Nevertheless, the primary author searched Google to access information from official health sources or pages supporting patients with cancer, as well as other articles that may contribute to the objective of the narrative literature review.

#### 2.1.4. Search Strategy

The search strategy for this study included the following keywords: “patients”, “caregivers”, “family members”, “cancer”, “assertive communication”, “extended family”, “communication”, “dyads”, “effective communication”, “communication guide”, “communication intervention”, “communications skills”, “relatives”, “aunts”, “uncles”, “cousins”, “grandparents”, “friends”, and “communication strategies”. A Boolean search was used to provide a systematic approach to searching that utilized keywords and logical operators (AND, OR) to refine and filter results, ensuring more accurate and relevant outcomes. For example, we used the operator “AND” to combine key terms such as “patients AND caregivers AND extended family AND communication”, while “OR” was used to include one of two terms like “aunts OR uncles” and “relatives OR extended family”. In addition, we conducted Google Scholar searches without using Boolean operators to ensure a broader retrieval of articles. We only included articles published between 2010 and 2024 and did not apply any date limitation to the online sources, since most did not have publication dates.

#### 2.1.5. Selection Process

To select the articles, the primary author (S.D.T.-M.) initially reviewed the articles’ titles in each database that might meet our criteria. The research team (S.D.T.-M., C.A.-B. and N.R.-T.) then conducted a three-step screening process to rigorously evaluate the article’s eligibility and ensure its content aligned with our review objectives. First, we conducted a title screening to review each title, identifying potential matches with our inclusion criteria. We moved the article titles that passed the first screening to the second screening. Second, we conducted an abstract screening, reviewing the abstracts to further determine eligibility. Finally, the abstracts that passed the second screening went to a full-text screening. The full-text screening included reviewing the entire article to ensure it met the inclusion or exclusion criteria. The research team held a consensus meeting for this final screening to discuss each article.

**Figure 1:**
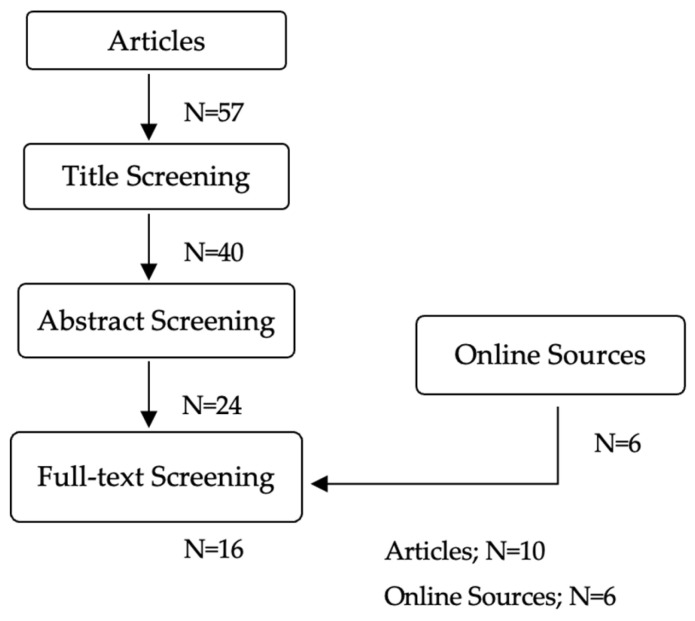
Flowchart of article selection process.

Additionally, we reviewed online sources, including websites of official health (e.g., the National Cancer Institute) and cancer support organizations (e.g., Pancreatic Cancer Action Network) that provide guidelines and recommendations on communication for patients and families. The primary author manually searched the online sources and selected possible informational material or pamphlets by title. Then, the research team (S.D.T.-M., C.A.-B. and N.R.-T.) conducted a full-text screening, reviewing their content to determine their eligibility based on the inclusion and exclusion criteria. Figure 1 shows a flowchart that includes numeric details of the selection process.

#### 2.1.6. Data Analysis

After selection, the research team (S.D.T.-M., C.A.-B. and N.R.-T.) read and analyzed the selected articles. The team summarized each article in an Excel sheet by title, author, year of publication, objective, study design, sample, method, results, and relevant conclusions. The research team created categories based on strategies and interventions that improve and promote effective communication between patients, caregivers, and extended family members.

### 2.2. Data Triangulation

We conducted a data triangulation based on the primary study findings, the narrative literature findings, and the cultural expert’s expertise, allowing us to develop the communication module focused on the extended family. Data triangulation combines multiple sources, such as data, methods, or theoretical perspectives, to cross-check, compare, and validate findings [18,19]. Using a data triangulation analysis allowed us to analyze and compare our findings, increasing the credibility of the research results [20].

#### 2.2.1. Primary Study

The primary study was conducted to develop a communication intervention for patients and caregivers coping with cancer. The study team explored the acceptability and facilitators of and barriers to developing a Communication Skills Training (CST) program guided by the Community Engagement Research (CER) framework [21,22]. The primary study was held in Puerto Rico, where the research team conducted semi-structured interviews with three focus groups. The focus groups comprised patients (with an active cancer diagnosis or with cancer recurrence), caregivers (any participant identified by the patient as their caregiver), community leaders (any community leader from the southern area of Puerto Rico), and healthcare providers (clinical psychology, social workers, physicians, and nurses managing more than 20 patients coping with cancer), all of any sex and aged 21 years or older. The primary study was conducted under ethical approval from the Ponce Research Institute Institutional Review Board (IRB #2206107691A002), ensuring compliance with ethical standards. The focus group interviews were recorded and transcribed. The research team developed the intervention through a qualitative thematic analysis of semi-structured interview transcripts using NVivo v12 (2020). The semi-structured interview protocol guided the thematic content analysis. More details regarding the primary study, such as participant characteristics, the semi-structured interview protocol, and other aspects of the methodology, can be found in the primary study [17], which was designed to gather comprehensive insights into the participants’ needs for the CST program. This primary study was not intended to include extended family members.

#### 2.2.2. Study and Cultural Expert Background

The primary study was held in Puerto Rico, a U.S. territory with a Hispanic and Latino population. Like many Hispanic and Latino cultures, Puerto Rican culture is deeply rooted in cultural values such as familism, collectivism, and strong intergenerational ties [23]. These cultural values may influence communication between patients, caregivers, and extended family members. Given the significance of familism in Puerto Rican and other Hispanic and Latino cultures, we included the expertise of a cultural expert (N.T.-B.) to conduct the data triangulation. The study benefited from the cultural expert’s knowledge of cultural practices, beliefs, and communication patterns relevant to the study context. Additionally, the expert of this study (N.T.-B.) has experience adapting psychosocial interventions such as the Caregiver–Patient Support to Latinx Coping with Advanced Cancer (CASA) intervention—a Hispanic and Latinos adaptation of Meaning-Centered Psychotherapy (MCP) and Couple Communication Skills Training (CCST) for patients and caregivers coping with advanced cancer [24,25].

#### 2.2.3. Data Triangulation Analysis

**Figure 2:**
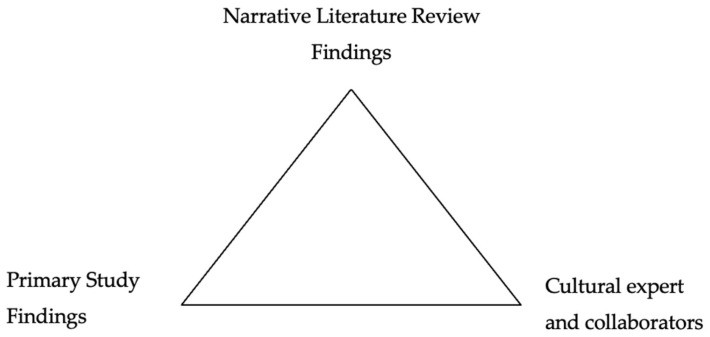
Sources of data triangulation.

We conducted a data triangulation analysis to integrate the primary study findings with the narrative review findings regarding the knowledge of the cultural expert. To perform the analysis, we used the primary study category findings, which included learning what to say, improving general communication, and treatment and symptoms [17]. These categories serve to triangulate the results with the narrative review findings. To conduct the data triangulation, the cultural expert (N.T.-B.) and collaborators (S.D.T.-M., C.A.-B. and N.R.-T.) compared the categories of the primary study with the narrative review findings. The cultural expert ensured that the communication strategies identified in the narrative review were aligned with the participants’ experiences and needs, as reported in the primary study. Using the information generated in the triangulation, we developed a module for patients and caregivers to communicate with extended family members. See Figure 2 for the overall view of the data triangulation sources.

## 3. Results

### 3.1. Narrative Literature Review Findings

The findings of the narrative literature review encompass strategies that promote effective communication among patients with cancer, caregivers, and extended family members. This NLR included 16 texts that passed the full-text screening. We categorized the findings according to the type of study, which included online sources (*n* = 6), preliminary studies (*n* = 6), randomized control trials (*n* = 2), and reviews (*n* = 2).

#### 3.1.1. Online Sources

The category of online sources refers to guidelines or webpages that provide communication strategies for patients with cancer from official cancer health websites or cancer support pages. We identified the subcategories of delegating (*n* = 4), prompt list (*n* = 4), methods of telling others (*n* = 3), creating a priority list (*n* = 2), being an active speaker (*n* = 2), being an active listener (*n* = 2), seeking support (*n* = 1), and using informational material (*n* = 1). Table 1 presents all texts categorized under online sources. Most online sources recommend delegating the task of sharing the cancer news to someone else, as it could be emotionally exhausting. Delegating is not just delivering the news about the illness but also helping manage others’ reactions [26,27,28,29].

Online sources mentioned preparing to talk about specific topics by writing key points or planning what to say when deciding what to communicate [26,28]. Also, it was recommended to discuss specific topics such as the name of the cancer, symptoms, treatment options or side effects and the patient’s feelings, expectations, needs, and hopes [27,28,30]. Regarding the methods of telling others, they described options such as in-person, phone calls, emails, text messages, or social media, depending on the closeness of the family member with the patient [26,29]. Harpham recommends using email because patients can update more than one person at a time instead of phone calls, which may be exhausting for the patient [27].

Additionally, it is important to consider with whom the patient is communicating. For this, creating a priority list, starting with the ones with whom the patients want to share the news first, can be helpful [29]. Also, by doing this, patients should think about the reaction they expect from that person, to prepare themselves for disclosing the news [26]. Since revealing a cancer diagnosis could be difficult for the patients, it is necessary to seek support from others, such as someone to accompany and encourage them during conversations with family [28]. Conversely, one helpful strategy for communicating with extended family is using and sharing informational material, which can help to reduce questions and provide a clear understanding of the condition [28].

Also, online sources offer recommendations for maintaining an effective conversation. Recommendations include being patient with oneself and others, being honest, and not hiding one’s feelings and emotions [31]. Additionally, it is suggested not to have any expectation of others’ reactions, since the cancer news could impact them. Both patients and extended family members are encouraged to be good listeners and ask genuine questions to maintain proactive and assertive communication [28].

**Table 1:** Finding of the online sources category.

Author (Year)	Title	Finding
Breast Cancer Foundation NZ (n.d.) [29]	Communicating to friends & family	Provides support for patients with cancer on how and what they could communicate with others.
City of Hope (n.d.) [31]	Communicating with friends and family during and after cancer treatment	The source provides tips or recommendations for communicating a cancer diagnosis to family and friends.
Harpham (2021) [27]	Managing communications with family & friends	A column blog that provides insights and tips, such as possible topics, for communicating with family and friends.
Mapes (2016) [26]	‘Coming out’ with cancer: Patients, experts discuss ins and outs of sharing a diagnosis	A personal experience of communicating with family members and provided tips to communicate with them.
National Cancer Institute (2015) [30]	Talking to family and friend about your advanced cancer	This source recommends approaching different family members and what the patient should expect from them.
Pancreatic Cancer Action Network (n.d.) [28]	Talking to your family about pancreatic cancer	This source provides recommendations for possible topics for communicating with different family members.

#### 3.1.2. Preliminary Studies

Preliminary studies refer to articles that employ a qualitative or quantitative approach, providing findings or results that refine or establish an initial foundation for subsequent studies. The subcategories of methods of telling others (*n* = 3), scheduling (*n* = 2), delegating (*n* = 2), seeking support (*n* = 2), a prompt list (*n* = 2), a priority list (*n* = 1), using informational material (*n* = 1), being an active listener (*n* = 1), and being an active speaker (*n* = 1) were identified. Table 2 reports the findings from the preliminary studies category. Most articles focus on various methods of telling others about the cancer diagnosis, which often depends on the person, the context, and what will be disclosed [32,33]. Participants in these studies reported that the most frequent or preferred method of disclosure was in person [32,33,34]. Even so, phone calls, messages, or emails were also used, especially for distant family members or when the news had to be communicated to multiple people simultaneously [32,33]. On the other hand, social media were used for updates about the diagnosis and when participants wished to avoid many explanations [32,33].

When disclosing a cancer diagnosis to extended family members, patients consider the timing for sharing the news. Tsuchiya et al. reported that patients preferred to share the cancer news upon the diagnosis, motivated by the need to receive emotional or practical support [34]. Nonetheless, Ewing et al. presented that the timing for disclosure depends on the patient’s readiness; some can share immediately, and others need more time to share [32]. Regarding readiness, patients might delegate the responsibility to share the news of the cancer diagnosis to someone else, but the patients usually decide whom to tell and when [32]. For this instance, Petersen et al. reported that more third-generation family members were informed about positive Lynch syndrome results from other relatives than the proband [35].

Having support is crucial, as encouragement to communicate about the cancer diagnosis plays a significant role [36]. This encouragement to speak about cancer or the family health history may be predicted by the patient’s self-efficacy, family cohesion, and family frequency of communication. Related to this, Haaksman et al. noted that encouraging patients and their relatives to communicate assertively facilitated a valuable conversation and promoted open communication [37]. Conversely, in this subcategory, patients also decide whom to communicate with based on priority, typically choosing those closest to them first. However, Fischer and Seibaek reported that patients also shared with others, like cousins, neighbors, and other patients coping with cancer, recognizing their essential role in the patient’s social network support [33].

Patients often feel insecure about what to say regarding their diagnosis. The findings encompassed that most patients shared news with family, yet the level of detailed information varied depending on the person. For example, some patients only shared a favorable prognosis or limited information, while others, by making updates, brought more detailed information [32,33]. Providing or using informational material to explain the diagnosis was another useful communication strategy for helping family members understand the diagnosis [35]. Also, patients had to deal with others’ reactions, such as extreme emotional responses. Concerning others’ reactions, participants in this study reported that being gentle and considerate was the most appropriate method for delivering the news, as it involved caring about the other person’s reaction [32].

**Table 2:** Findings of the preliminary studies category.

Author (Year)	Title	Study Type	Sample	Findings
Ewing et al. (2016) [32]	Sharing news of a lung cancer diagnosis with adult family members and friends: A qualitative study to inform a supportive intervention	Qualitative study	Phase 1: 20 patients, 17 accompanying individuals, 27 healthcare professionals. Phase 2: 24 healthcare professionals and six service users	This study identifies the challenge of sharing bad news and a potential framework to guide the delivery of a supportive intervention tailored to patients’ individual needs.
Fisher and Seibaek (2021) [33]	Patient perspectives on relatives and significant others in cancer care: An interview study	Qualitative study	17 women in gynecological cancer treatment	Relatives represent a unique resource and support to patients. Additionally, neighbors and people who had experienced cancer were an important and valuable support to the patients.
Haaksman et al. (2024) [37]	Open communication between patients and relatives about illness & death in advanced cancer—results of the eQuiPe Study	A prospective, longitudinal, multicenter, observational cohort study	160 bereaved relatives of patients with advanced cancer	Open communication about illness and death between patients and relatives seems to be important, as it is associated with a lower degree of bereavement distress.
Peterson et al. (2018) [35]	Patterns of family communication and preferred resources for sharing information among families with a Lynch syndrome diagnosis	Qualitative study	127 participants: 32 probands (individuals identified with the mutation) and 95 family members	Both probands and family members were most likely to share genetic test results with parents and siblings and least likely to share the results with aunts, uncles, and cousins.
Rodríguez et al. (2016) [36]	Family ties: The role of family context in family health history communication about cancer	Correlational study	472 women	Greater family cohesion, flexibility, and a higher self-efficacy were related to a higher communication frequency and cancer information sharing.
Tsuchiya et al. (2022) [34]	Cancer disclosure to friends: Survey on psychological distress and perceived social support provision	Correlational study	473 patients with cancer	A more significant pre-disclosure distress was associated with being a young adult, being a woman, and delaying disclosure. After disclosing, participants perceived receiving emotional support.

#### 3.1.3. Randomized Control Trials

The randomized control trials (RCTs) category included studies that measured interventions with a communication component among patients, caregivers, and family members. We included the following subcategories: coaching and educating on communication skills and assertive communication (*n* = 2), practicing (*n* = 2), and using booklets (*n* = 1). Table 3 presents the articles included in the category of RCTs. As part of the coaching and educating component, Bodurtha et al. coached participants on communicating with family members to obtain information about the family cancer risk history [38]. Similarly, Chiquelho et al. included a communication component in their psycho-educational approach intervention by educating participants on various communication methods [39]. These methods included passive, aggressive, manipulative, and assertive communication, emphasizing the importance of the last one. For assertive communication, this intervention employed the DESC technique, an acronym for describing the facts, expressing feelings, specifying the desired change, and explaining the consequence.

Additionally, the interventions included using a booklet to guide and demonstrate to participants how to become assertive communicators by being active listeners and aware of their body language [38]. It also provided a question guide to help participants decide with whom, when, and where to communicate with a family member [38]. Finally, the RCTs included a practicing component, where participants were encouraged to provide real-life examples to apply the learned communication skills and practice them in their lives, with feedback provided afterward [38,39].

#### 3.1.4. Reviews

The review category includes both narrative and systematic literature reviews. Within this category, the articles addressed prompt lists (*n* = 1), being an active listener (*n* = 1), and being an active speaker (*n* = 1). Wallace includes possible topics or prompt lists to guide conversations between patients and family members [40]. These possible topics included the quality of the relationship, personal and relational identities, routine interactions, memories, plans for the future, problem-solving, and messages about love, faith, gratitude, forgiveness, and farewells [40]. On the other hand, for being an active listener, patients are encouraged to consider the perspective of the person they are speaking with. Kishino et al. emphasized the importance of considering the other person’s point of view, including the need to clarify and encourage questions from others [12]. Finally, as part of being an active speaker, a review provided a communication component in a potential advance care planning (ACP) intervention, highlighting the importance of allowing time for honest and respectful conversations between patients and family members [12]. See Table 4 for the findings of the review category.

In summary, we divided the NLR findings into four main categories based on the type of articles or sources. The four main categories were online sources, preliminary studies, RCTs, and reviews. These categories were divided by the communication strategies identified within the content of the articles and online sources. A prompt list (*n* = 7), delegating (*n* = 6), and methods of telling others (*n* = 6) were the most identified within the communication strategies. Also, they included being an active listener (*n* = 4), being an active speaker (*n* = 4), creating a priority list (*n* = 3), and seeking support (*n* = 3) as strategies that will enhance communication among patients, caregivers, and extended family. Finally, we identified scheduling (*n* = 2), using informational material (*n* = 2), coaching and educating (*n* = 2), practicing (*n* = 2), and using booklets (*n* = 1).

### 3.2. Integration of the Findings to Protocol

We conducted a data triangulation to integrate the narrative review findings with focus group findings from the primary study. The most accepted content of communication with extended family was learning what to say, improving general communication, and treating symptoms [17]. The cultural expert (N.T-B.) and collaborators (S.D.T.-M., C.A.-B. and N.R.-T.) conducted the integration of the narrative findings to develop the extended family module in Table 5. By identifying the communication strategies and findings of the focal groups, we incorporated possible topics and other strategies to guide the patient and caregiver on what to communicate to the extended family. The learning what to say content includes the following: (1) prompt list, (2) delegating, (3) methods of telling others, (4) priority list, (5) seeking support, (6) using informational material, (7) training and educating, (8) booklets, and (9) practicing. The treatment and symptom content were included within the prompt list strategy. Additionally, we included guidelines on general assertive communication skills for both speaker and listener when talking to extended family members. The improving general communication content includes (1) active listener: patience and honesty, (2) active speaker: patience and honesty, (3) training and educating, (4) booklet, and (5) practicing.

## 4. Discussion

This study aimed to develop a communication module to enhance communication among patients and caregivers coping with cancer and their extended family members. We designed the communication module by integrating two sources (a narrative literature review and primary study findings) and a cultural expert’s expertise through data triangulation. Based on the data triangulation findings, the study team developed a communication module tailored to patients with cancer and caregivers’ needs. Given our findings, the communication module includes two main components: learning what to say and improving general communication. These components were developed to address the needs of patients and caregivers coping with cancer, while considering the cultural values that shape family interactions in Hispanic and Latino communities.

The content of learning what to say was developed by incorporating various communication strategies identified in the narrative review, such as a prompt list, delegating, methods of telling others, and creating a priority list, among others. The prompt list strategy, the most highlighted strategy, was incorporated by providing potential topics that help patients and caregivers converse with extended family. Potential topics suggested in the literature were disclosing the cancer diagnosis, symptoms, treatment, and prognosis [17,36]. Moreover, delegating emerged, since the patients may need another person (i.e., a caregiver) to share the news; in this instance, the caregiver should learn what to say. Additionally, deciding what to say may be influenced by the communication method and the person, the strategies of methods of telling others (i.e., in person, by phone), and a priority list was included. A priority list allows both patients and caregivers to decide which family member they are going to talk to when delivering the news of a cancer diagnosis for the first time [32,38]. In line with this, selecting disclosure is crucial in reducing psychosocial stress and increasing perceived social support [34]. Incorporating these communication strategies to learn what to say may help patients and caregivers navigate the cultural expectation of familism.

The components of improving general communication skills included strategies such as being an active listener and speaker. We must consider patience and honesty as part of both communication strategies, which are vital to assertiveness. Improving general communication assertively is essential, despite the sensitive context of coping with a cancer diagnosis [41]. Moreover, effective communication can enhance emotional well-being, relationships, and interactions [42]. These findings reinforced the inclusion of these strategies in the module content to improve patient, caregiver, and extended family communication.

The team ensured the implementation of the communication strategies by including education, training, booklet use, and practice strategies. Education provides patients and caregivers with an understanding of communication strategies, while training and practice allow participants to apply the taught strategies in real and hypothetical scenarios. Studies demonstrate that training participants in communication enhances their ability to communicate better with relatives, supporting the inclusion of this strategy in the module [34,36,38,39].

Developing this communication module addresses a critical gap, accounting for the crucial role of cultural factors in shaping communication between cancer patients and their families within Puerto Rican and broader Hispanic and Latino communities. One of these cultural factors is familism, which emphasizes interdependence, family cohesion, and collective decision-making, which can enhance support systems [8,9,11]. Familism served both as a central rationale for its development and as a guiding principle in the module’s integration and content, aiming to address communication needs and barriers specific to these cultural values.

Conducting cultural adaptations is essential for addressing the needs of specific cultural backgrounds, such as in Puerto Rico. Culturally adapted interventions targeting a specific cultural group are four times more effective than those provided to various cultural backgrounds [43,44]. In this study, the cultural expert has a vital role in guiding the development of the communication module, especially given that all the studies identified in the narrative literature review were not focused on Hispanic and Latino communities. Although the module was specifically tailored to the Puerto Rican context, its findings may also be relevant to other Hispanic and Latino communities due to shared cultural values such as familism. However, it is crucial to recognize that cultural adaptations should consider varying levels of familism and specific community dynamics within different Hispanic and Latino groups to ensure their effectiveness and cultural responsiveness [43,44].

The findings of this study have important implications for healthcare professionals, research, and communities. Recognizing and considering cultural values will allow healthcare professionals to navigate family dynamics in decision-making, address potential communication barriers, and support patient autonomy within culturally appropriate frameworks. Additionally, implementing the communication module in communities can foster stronger support networks, promote trust in healthcare providers, and ensure that interventions are relevant and effective in the context of specific cultural beliefs and practices [45,46]. Moreover, culturally grounded implementation research may improve the adoption and impact of interventions [47,48], leading to improved communication between patients and family members during the cancer care trajectory.

This study has some limitations. The narrative literature review revealed limited studies or articles that address communication strategies that target communication between patients, caregivers, and extended family members. Additionally, the primary research was not to design a communication module focused on extended family. Despite these limitations, the research team incorporated diverse sources in the narrative review, such as RCTs and online sources, to provide a comprehensive perspective for the communication module’s development. Finally, the team integrated the primary study and narrative review findings into the data triangulation, which ensures that the module addresses the needs of the primary study participants.

## 5. Conclusions

Integrating extended family members into the patient’s coping process with cancer is often crucial in Hispanic/Latino communities. The NLR results and data triangulation highlight an essential step toward refining and adapting the module. By developing the communication module, we deliver training and improve communication among patients with cancer, caregivers, and extended family members. Overall, future studies should explore the acceptability and implementation strategies of protocols focused on communication.

## Figures and Tables

**Table 3:** Findings of the randomized control trials category.

Author (Year)	Title	Type of Study	Sample	Findings
Bodurtha et al. (2014) [38]	The KinFact intervention: A randomized controlled trial to increase family communication about cancer history.	RCT	490 women; 245 per group	The KinFact intervention successfully promoted family communication about cancer risk by educating women to enhance their communication skills surrounding family history.
Chiquelho et al. (2011) [39]	Pro families: a psycho-educational multifamily group interventions for cancer patients and their families.	Quasi-experimental study	57 participants from 19 families, divided into five groups	The program responds to the patients’ and families’ needs, prevents an increase in the patient’s level of psychosocial maladjustment, promotes an adequate level of family cohesion, and diminishes the perceived stress of patients and family members.

**Table 4:** Findings of the review category.

Author (Year)	Title	Type of Study	Sample	Findings
Kishino et al. (2022) [12]	Family involvement in advance care planning for people living with advanced cancer: A systematic mixed-methods review	Systematic mixed-method review	14 articles	This review identified individuals’ and family members’ perceptions concerning family involvement in advance care planning and presented components for a family-integrated advance care planning intervention.
Wallace (2014) [40]	Family communication and decision making at the end of life: A literature review	Narrative literature review	This review did not include the sample size.	Family members’ communication is crucial during end-of-life care.

**Table 5:** Extended family communication module that was culturally adapted and integrated the narrative review findings.

Communication Strategies	Content	Adaptation
Prompt list Delegating Methods of telling others Priority list Seeking support Using informational material Training and educating Booklet Practicing	Learn what to say	We included possible topics (e.g., disclosing the cancer diagnosis, talking about the prognosis of the illness, treatment and symptoms, and sharing emotions and thoughts) and strategies (e.g., delegating, considering the appropriate methods for delivering information, and creating a priority list of family members) to guide the patient and caregiver on what to communicate to extended family.
Active listener: Patience Honesty Active speaker: Patience Honesty Training and educating Booklet Practicing	Improve general communication	We included guidelines on general assertive communication skills as a speaker and listener.

Note: The treatment and symptoms content are included in the communication strategies: prompt list.

## Data Availability

Data can be shared upon request.
